# Social alienation among people with bladder cancer: A combined structural equation modeling and fuzzy-set qualitative comparative analysis

**DOI:** 10.1016/j.apjon.2026.101025

**Published:** 2026-09-11

**Authors:** Yixuan Yang, Lu Han, Jie Li, Jun Xue, Ting Wang, Qiuzhi Zhao, Cui Xie, Jiaping Wang, Xue Wang, Li Fu

**Affiliations:** a The Second Hospital of Tianjin Medical University, Tianjin, China; b School of Nursing, Tianjin University of Traditional Chinese Medicine, Tianjin, China

**Keywords:** fsQCA, SEM, Bladder cancer, Social alienation, Psychological distress

## Abstract

**Objective:**

People with bladder cancer may experience social alienation, which is associated with adverse psychosocial outcomes. This study investigates the complex factors and configurations associated with this condition.

**Methods:**

In this single-center cross-sectional study, 260 patients with bladder cancer from a Chinese tertiary hospital completed measures of family resilience, peer support, psychological distress, negative social expectations, and social alienation. Data were analyzed using structural equation modeling (SEM) and fuzzy-set qualitative comparative analysis (fsQCA).

**Results:**

SEM showed that family resilience (Estimate = −0.337, *P* = 0.001) and peer support (Estimate = −0.284, *P* = 0.022) were negatively associated with social alienation, whereas psychological distress (Estimate = 0.137, *P* = 0.049) and negative social expectations (Estimate = 0.278, *P* = 0.004) were positively associated with social alienation. Psychological distress and negative social expectations contributed to several indirect associations. The fsQCA identified no single necessary condition (all necessity consistencies < 0.90) and six configurations associated with high social alienation (overall solution consistency = 0.911, overall solution coverage = 0.54; configuration consistencies = 0.951–0.96), grouped into two broad profiles characterized by limited socioeconomic resources with negative social expectations, or psychological distress with limited expressive peer support.

**Conclusions:**

Social alienation among patients with bladder cancer is characterized by multiple combinations of interpersonal and psychological conditions. These findings may inform profile-based assessment and the development of supportive-care hypotheses, but longitudinal and intervention studies are required before causal or treatment conclusions can be drawn.

## Introduction

Bladder cancer (BCa), as the most prevalent malignancy of the urinary system, poses a significant global health burden with rising incidence and mortality.^1^ For patients, the disease trajectory extends far beyond managing physical symptoms like hematuria and bladder irritation.^2^ Critically, the high recurrence rate, reaching up to 70% for non-muscle-invasive BCa, necessitates lifelong, intensive surveillance, repeated treatments, and frequent hospitalizations.^3^ This extended disease course imposes substantial financial toxicity, with patients facing out-of-pocket costs and productivity losses,^4^ while simultaneously generating profound psychological strain marked by uncertainty, anxiety, and fear of recurrence.^5^ This combination of factors is associated with a heightened risk of social alienation, a debilitating state of disconnection characterized by a lack of interpersonal support, loss of social channels, and pervasive negative emotions.^5^^,^^6^

Social alienation is not merely a concomitant symptom but a critical stressor and factor associated with adverse outcomes in chronic illness populations. As evidenced in head and neck cancer, prostate cancer, and inflammatory bowel disease patients, changes in body image, functional disabilities (e.g., urinary incontinence), and the self-perceived burden on others may be associated with profound estrangement and avoidance of social life.^7^^,^^8^ The consequences are severe, encompassing loneliness, depression, diminished treatment compliance, and an elevated risk of mortality.^9^, ^10^, ^11^ Several risk and protective factors have been implicated in this process. Regarding risk factors, a recent systematic review^12^ specifically highlights that psychological distress, encompassing anxiety and depression, is highly prevalent in bladder cancer patients and is exacerbated by a lack of social support. Similarly, negative social expectations, pessimistic beliefs about one's social value and relationships, have been shown to be significantly associated with loneliness among patients with cancer.^13^ Conversely, emerging evidence points to protective factors that may buffer against alienation. Family resilience, the family's capacity to withstand and adapt to adversity, is associated with better psychosocial outcomes for both adult cancer patients and their family members.^14^ Peer support, including emotional expression and illness perception, has similarly demonstrated protective effects against distress and isolation in cancer populations.^15^ However, despite its clinical significance, social alienation and its underlying factors and patterns in the BCa population remain markedly under-explored, creating a glaring gap in supportive care.

The Cognitive-Affective Personality System (CAPS) theory provides a robust framework for deconstructing this complexity by delineating how stable patterns of behavior arise from interactions between the situation and an individual's internal cognitive-affective units.^16^ Applied to BCa, the interpersonal context, encompassing family resilience (the family's capacity to withstand and adapt to adversity) and peer support can be conceptualized as key situational features that shape a patient's social environment.^9^^,^^17^ These situational features are thought to be associated with critical internal cognitive-affective units: negative social expectations (pessimistic beliefs about one's social value) and psychological distress (anxiety, depression). In turn, these internal states are hypothesized to be associated with social alienation, potentially accounting for indirect relationships between interpersonal context and alienation.^8^^,^^10^^,^^18^ While this CAPS-informed model is plausible, its empirical validation in BCa is absent, and the methodological approaches in existing psychosocial oncology research often fail to capture the full picture.

Prevailing research predominantly relies on variable-centered methods like Structural Equation Modeling (SEM).^19^^,^^20^ While SEM is powerful for testing the net association of isolated variables and pre-defined indirect paths, it is inherently limited by its symmetric logic and inability to capture complex configurational associations. A critical, overlooked reality is that patients present with complex, multifaceted profiles where different combinations of conditions are associated with the same outcome, a principle known as equifinality.^21^ Theoretically, equifinality is highly expected in social alienation, a multifaceted outcome. For instance, one patient might exhibit high alienation in the context of financial hardship and lack of practical resources (an ‘accelerated disability’ pathway), while another might similarly exhibit high alienation in the context of overwhelming psychological distress coupled with an inability to express their feelings to peers, despite maintaining a positive outlook (a ‘cognitive dissonance’ pattern). Traditional correlational and mediation analyses are ill-equipped to answer questions about the configurational patterns associated with social alienation, leaving a significant methodological gap that hinders the development of personalized interventions.

Our study introduces a multi-method analytical approach that integrates SEM with fuzzy-set Qualitative Comparative Analysis (fsQCA). In practical terms, while SEM explains whether factors like family resilience have a statistically significant association with social alienation, fsQCA identifies which specific combinations of factors (e.g., high psychological distress and low expressive peer support) consistently characterize patients who experience high alienation. This synergistic design serves two complementary objectives. The first is to examine whether the associations of interpersonal resources (family resilience and peer support) with social alienation are partially accounted for by critical cognitive-affective states (psychological distress and negative social expectations). The second is to explore the complex, equifinal nature of social alienation by identifying which specific combinations of these risk and protective conditions are consistently associated with high levels of the outcome. Achieving these aims will provide a more nuanced, clinically actionable understanding of social alienation, moving beyond one-size-fits-all explanations to acknowledge the diverse lived experiences of patients.

## Methods

### Participants

This cross-sectional study was conducted between November 2023 and June 2024 using convenience sampling. Participants were recruited from the urology outpatient and inpatient departments of the Second Hospital of Tianjin Medical University in Tianjin, China. The inclusion criteria were: (1) a confirmed diagnosis of BCa, and (2) provision of informed consent by the patient. The exclusion criteria were: (1) Presence of any of the following severe comorbidities within 6 months prior to enrollment: ① Stroke with significant neurological deficit (modified Rankin Scale score ≥ 3); ② Heart failure with New York Heart Association (NYHA) functional class III–IV; ③ End-stage renal disease (chronic kidney disease stage 5, estimated glomerular filtration rate (eGFR) < 15 mL/min/1.73 m^2^ or receiving renal replacement therapy), as judged by the investigator to have no direct causal relationship with bladder cancer; (2) History of cognitive impairment or psychiatric disorders, as judged by the investigator to be unable to comply with study procedures.

The sample size was determined by considering the requirements of both analytical methods employed in this study. For SEM, a preliminary analysis of 100 randomly selected questionnaires using AMOS 23.0 identified 34 free parameters. Following the guideline that the sample size should be five to 10 times the number of free parameters, the target sample size was estimated to be between 170 and 340.^20^^,^^22^ For a fsQCA with k conditions, the minimum recommended sample size is 2^(*k*−1)^. With *k* = 9 conditions in our model, the required minimum is 2^(9−1)^ = 256 cases.^23^, ^24^, ^25^ The target sample size was set between 170 and 340. The final sample of 260 cases satisfies the requirements of both methods.

### Measures

In the pilot survey (*n* = 50), with the exception of the Distress Thermometer, which could not be assessed for reliability as a single-item observed indicator, the remaining scales yielded Cronbach's *α* coefficients ranging from 0.76 to 0.95, the KMO measure of sampling adequacy ranged from 0.787 to 0.890, and Bartlett's test of sphericity was significant (all *P* < 0.001), indicating acceptable to excellent internal consistency.

#### Family resilience

Family resilience was assessed using the culturally adapted Chinese version of the Family Resilience Assessment Scale.^26^ This 44-item instrument comprises four subscales: family communication and problem-solving, utilization of social and economic resources, maintaining a positive outlook, and ability to make meaning of adversity. In this study, the scale showed excellent psychometric properties, with a Cronbach's *α* of 0.877, KMO measure of 0.787.

#### Peer support

Peer support was measured by the Chinese version of the Cancer Peer Support Scale.^27^ The 11-item scale includes three dimensions: output expression, self-expression, and illness perception. In this study, it demonstrated good internal consistency, with a Cronbach's *α* of 0.87, KMO measure of 0.824.

#### Psychological distress

Psychological distress was evaluated using the Distress Thermometer, a single-item self-report tool developed by the National Comprehensive Cancer Network.^28^ Patients rated their average distress over the past week on a scale from 0 (no distress) to 10 (extreme distress). The DT has proven validity in screening for cancer-related distress.^29^

#### Negative social expectations

Negative social expectations were assessed with the Chinese version of the Cancer-Related Negative Social Expectations Scale, originally developed by Adams et al.^30^ This 5-item instrument uses a 6-point Likert scale (scores range from five to 30), with higher scores indicating greater negative expectations. In this study, the Chinese version exhibited good internal consistency (Cronbach's *α* = 0.93, KMO = 0.890).

#### Social alienation

Social alienation was measured using the Chinese version of the General Alienation Scale.^31^ This 15-item scale covers four dimensions: self-alienation, distrust, interpersonal alienation, and sense of meaninglessness. Total scores range from 15 to 60, with higher scores indicating greater alienation. In this study, the scale showed acceptable reliability (Cronbach's *α* = 0.76, KMO = 0.838).

### Data collection

Prior to formal data collection, all research staff underwent standardized training to ensure consistency in procedures. Questionnaires were administered via paper-and-pencil format in quiet clinical settings when participants were mentally alert and had at least 30 minutes of uninterrupted time. For participants with visual impairments or limited literacy, trained staff provided one-on-one assistance by reading the items aloud and recording their responses. This study was conducted in accordance with the Declaration of Helsinki and approved by the Medical Ethics Committee prior to data collection. Initially, 302 patients were enrolled, 29 were excluded because they were unwilling to continue answering the questionnaire, and 13 were excluded due to patterned responses. According to the prespecified data-handling rule, questionnaires with <10% missing data would have been processed using multiple imputation, and those with >10% missing data would have been excluded as invalid. However, all included cases had complete data on the key variables; therefore, multiple imputation was not performed, and no cases were excluded on the basis of missing data. Ultimately, 260 patients completed the study, yielding a valid inclusion rate of 86.1%. A flowchart of the data collection process is presented in Fig. 1.

**Fig. 1:**
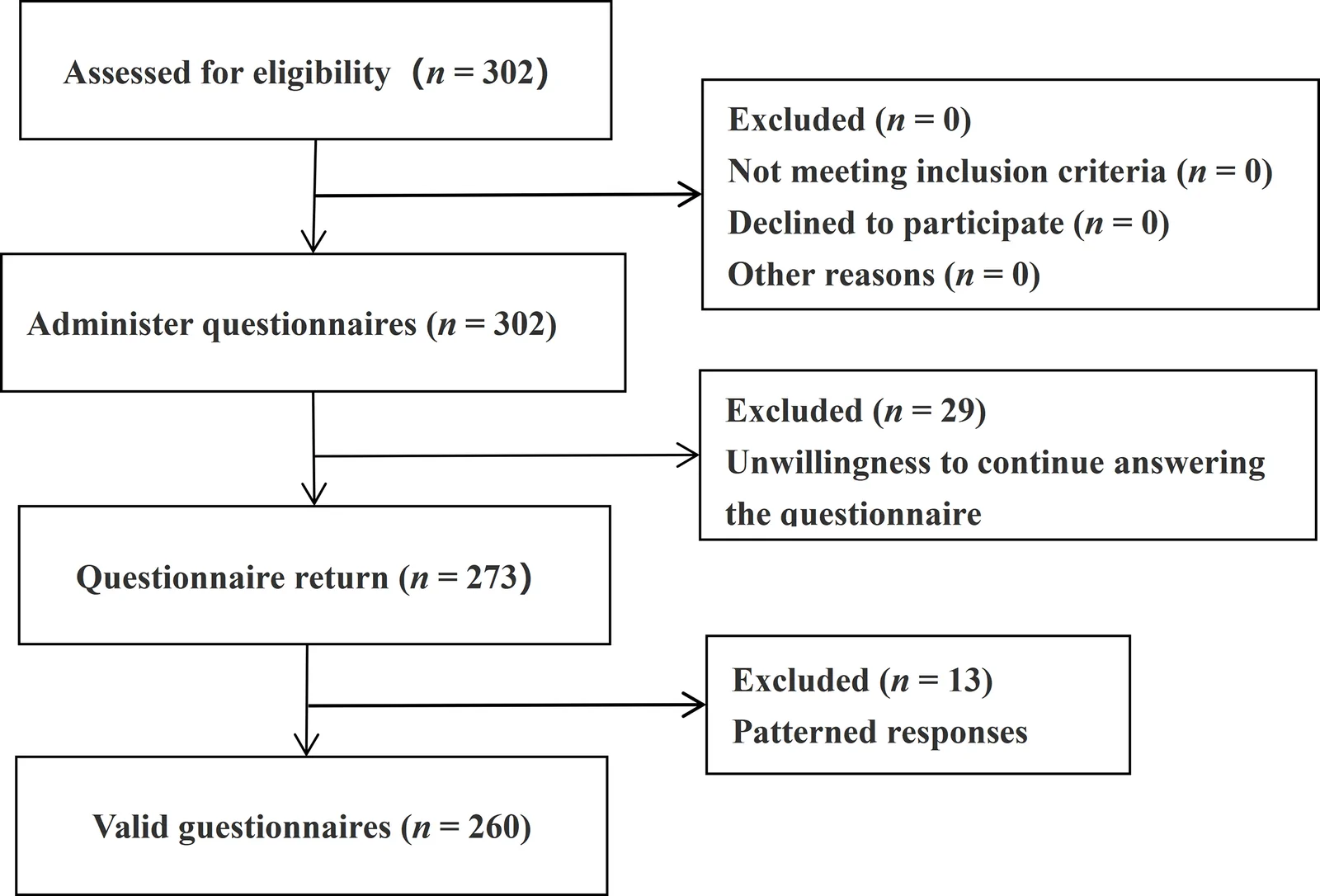
Flowchart of patient recruitment and survey procedures.

### Data analysis

Data analysis was conducted using SPSS Statistics version 27.0 (IBM, Inc., Chicago, IL, USA), AMOS 23.0, and fsQCA 3.0 software. A complementary dual-method analytical approach integrating both variable-oriented and case-oriented techniques was employed to comprehensively address the research hypotheses. The analysis proceeded in three sequential phases. In this framework, Family Resilience, Peer Support, Negative Social Expectations, and Social Alienation were modeled as latent variables, whereas Psychological Distress was treated as a single observed indicator.

#### Descriptive and univariate analysis

Normally distributed continuous data were reported as mean ± standard deviation (Mean ± SD), while non-normally distributed data were presented as median and interquartile range (Median [IQR]). Categorical variables were expressed as frequencies with percentages (%). To examine differences in social alienation scores across sociodemographic characteristics, independent samples t-tests were used for binary categorical variables. For categorical variables with three or more groups, one-way analysis of variance (ANOVA) was applied when the data met the assumptions of normality and homogeneity of variance; otherwise, the Kruskal–Wallis *H* test was used. To address potential common method bias in the results, Harman's single-factor test was applied. A *P*-value of less than 0.05 (two-tailed) was considered statistically significant.

#### Structural equation modeling

A SEM was constructed based on the CAPS theory^32^ to test the hypothesized direct and indirect associations. The CAPS framework posits that behavior arises from the interaction of the situation, cognitive-affective units, and behavioral responses. Accordingly, we hypothesized that: (1) Interpersonal context (family resilience and peer support) is directly associated with behavioral outcome (social alienation). (2) Cognitive-affective units (psychological distress and negative social expectations) are directly associated with social alienation, as potential internal factors associated with behavior. (3) The interpersonal context (e.g., family resilience) was hypothesized to be associated with cognitive-affective units (e.g., psychological distress), which in turn, are associated with social alienation. This is consistent with the CAPS theory's premise that the situation is linked to internal states, which then are linked to behavior.^8^^,^^10^^,^^18^ Sociodemographic characteristics (age, marital status) were included in the model as control variables. Model fit was assessed using multiple indices: chi-square/degrees of freedom (CMIN/DF), goodness-of-fit index (GFI), adjusted goodness-of-fit index (AGFI), comparative fit index (CFI), Tucker–Lewis index (TLI), and root mean square error of approximation (RMSEA). A model was deemed acceptable if CMIN/DF was between 1 and 3, GFI and AGFI > 0.80, CFI and TLI > 0.90, and RMSEA < 0.08.^33^ The significance of mediation effects was tested using bias-corrected bootstrap analyses with 5000 resamples and 95% confidence interval (CI).

#### Fuzzy-set qualitative comparative analysis (fsQCA)

FsQCA was used to explore how family resilience, peer support, psychological distress, and negative social expectations interact in complex configurations associated with social alienation. Both Family Resilience and Peer Support are multidimensional constructs. Reducing each to a single composite variable would inevitably obscure important nuanced information and fail to capture the differential effects of specific dimensions on the outcome. By treating each dimension as a separate causal condition, we are able to more finely identify which specific dimensions play core versus peripheral roles in which configurations, thereby yielding more granular and theoretically informative insights.^34^ This method identifies multiple configurations of conditions that are consistently associated with the outcome (equifinality). The analysis followed a standard three-step procedure.

(1) Calibration

All variables were transformed into fuzzy-set membership scores ranging from 0 to 1. Following standard fsQCA procedures, the 95th, 50th, and fifth percentiles of the sample distribution were used as anchors for full membership, the crossover point, and full non-membership, respectively. This data-driven approach was chosen to ensure the calibration was directly anchored to the empirical distribution of the sample.^24^

(2) Necessity analysis

We first tested whether any single condition was necessary for high levels of social alienation, using a consistency threshold of 0.9.

(3) Sufficiency analysis (Configurational Analysis)

A truth table was subsequently constructed, and a configuration analysis was performed to identify condition combinations associated with high levels of social alienation. The analysis yielded three types of solutions: complex, intermediate, and parsimonious solutions. Boolean minimization was used to combine intermediate and parsimonious solutions, enabling the identification of core and peripheral conditions, both in their presence and absence, characterizing different configurations.^35^

## Results

### Common method bias test

Harman’s single-factor test showed that a single factor accounted for 16.1% of the total variance, below the 50% threshold.

### Participant characteristics

A total of 260 patients with BCa were included in the final analysis. All included cases had no missing data on the key variables. The general characteristics of the participants and the univariate comparisons of social alienation scores across these factors are presented in Table 1.

**Table 1:** General characteristics of participants and distribution of social alienation scores (*N* = 260).

Characteristics		*n* (%)	Social alienation scores	Test statistic	*P* value
Sex	Female	52 (20.0)	33.77±7.30	−4.062^t^	< 0.001
	Male	208 (80.0)	29.55±6.54		
Age, years	18–59	44 (16.9)	31 (24, 35)	1.139^H^	0.566
	60–69	97 (37.3)	30 (23, 35)		
	≥ 70	119 (45.8)	31 (26, 35)		
Education level	Primary school	33 (12.7)	30 (24, 35)	1.978^H^	0.577
	Junior high school	86 (33.1)	30 (24, 34)		
	Senior high school	79 (30.4)	32 (26, 36)		
	Undergraduate	62 (23.8)	29.5 (24.0, 34.2)		
Marital status	Married	207 (79.6)	29.77 ± 7.06	−2.923^t^	0.004
	Other	53 (20.4)	32.83 ± 5.61		
Employment status	Not employed	215 (82.7)	29.55±6.54	1.114^t^	0.266
	Employed	45 (17.3)	29.36±7.46		

H Kruskal–Wallis *H.*

t Independent Samples *t* test.

### Descriptive statistics for study variables

Table 2 summarizes the descriptive statistics for all variables included in the SEM and qualitative comparative analysis, namely Family Resilience, Peer Support, Psychological Distress, Negative Social Expectations, and Social Alienation.

**Table 2:** Descriptive statistics for study variables (*N* = 260).

Latent variables	Mean	SD	Observed range	Skewness	Kurtosis
Family resilience	138.87	12.07	81–164	−0.988	2.623
Peer support	42.89	7.87	25–55	−0.302	−0.886
Psychological distress	2.80	2.31	0–9	0.504	−0.855
Negative social expectations	14.77	7.43	5–30	0.296	−1.185
Social alienation	30.40	6.90	15–60	−0.033	−0.440

SD, standard deviation.

### Structural equation modeling results

The final model demonstrated a good fit to the data: *χ*^2^/*df* = 2.218 (*χ*^2^ = 314.997, *df* = 142), GFI = 0.921, AGFI = 0.852, CFI = 0.921, TLI = 0.905, RMSEA = 0.069. The path coefficients, with covariates adjusted, are summarized in Supplementary Table S1 and depicted in Fig. 2, with covariate paths omitted for clarity. The inclusion of age and marital status as control variables did not alter the significance or direction of any hypothesized paths.

**Fig. 2:**
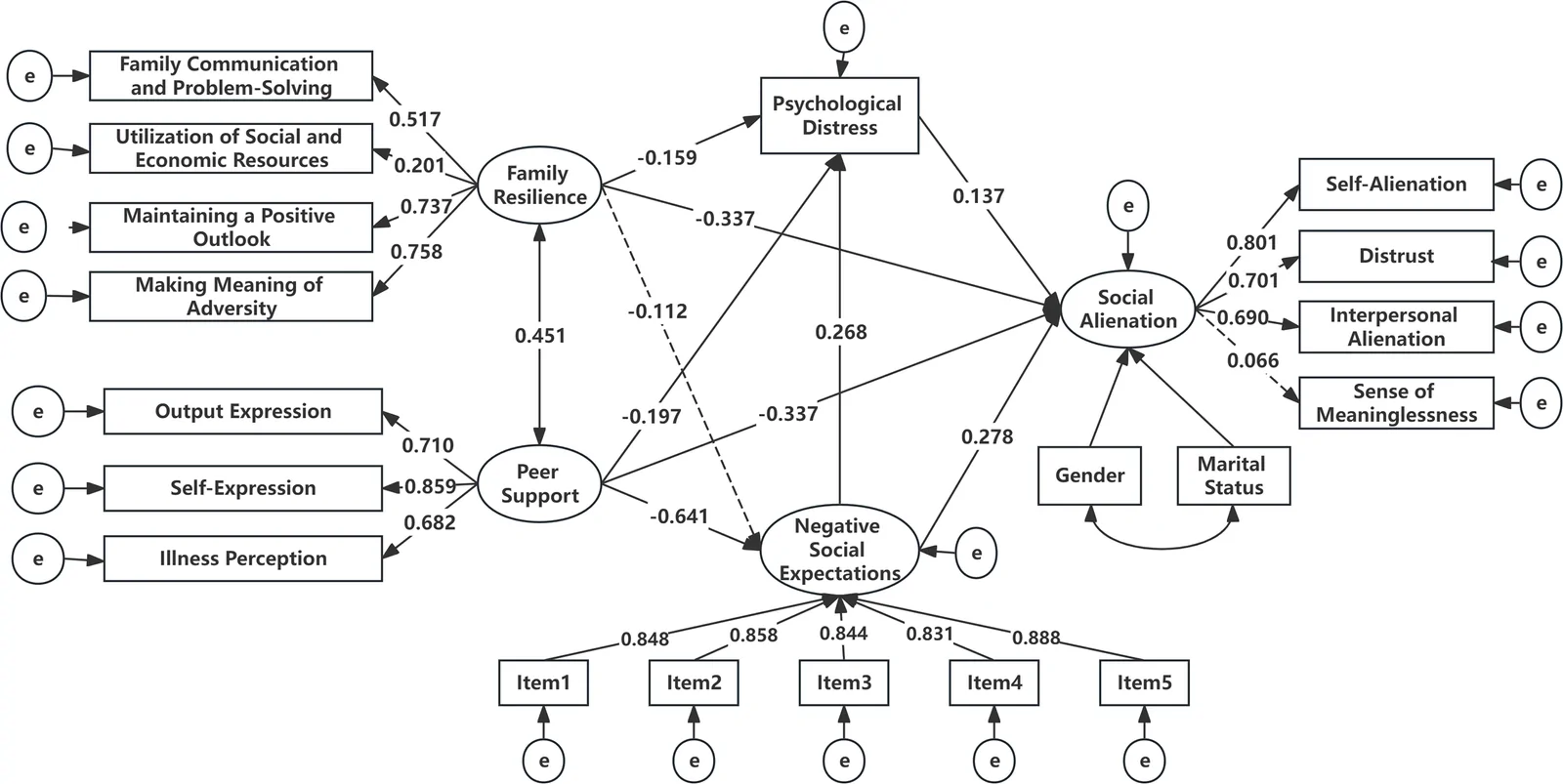
Results of the structural equation model.

Family resilience, peer support, and negative social expectations all showed significant direct associations with social alienation. Furthermore, psychological distress and negative social expectations accounted for significant indirect associations in the relationships. Specifically, psychological distress was observed to account for a significant indirect association between family resilience and social alienation, as well as between peer support and social alienation. Negative social expectations accounted for a significant indirect association between peer support and social alienation. However, the specific indirect association of family resilience with social alienation through negative social expectations was not statistically significant. Nevertheless, the total indirect association of family resilience with social alienation was significant, primarily reflecting the significant indirect association through psychological distress. The detailed results of the mediation analysis are presented in Table 3.

**Table 3:** Mediation effects test using bootstrapping.

Mediation path	Effect type	Estimate (95% CI)	*SE*	*P* value
Negative social expectations → psychological distress → social alienation	Total effect	0.315 (0.112, 0.513)	0.099	0.002
	Direct effect	0.278 (0.088, 0.418)	0.097	0.004
	Indirect effect	0.037 (0.003, 0.100)	0.024	0.031
Family resilience → social alienation	Total effect	−0.394 (−0.563, −0.220)	0.087	< 0.001
	Direct effect	−0.337 (−0.503, −0.167)	0.085	0.001
Family resilience → psychological distress → social alienation	Indirect effect	−0.022 (−0.063, −0.001)	0.015	0.038
Family resilience → negative social expectations → social alienation	Indirect effect	−0.031 (−0.082, 0.000)	0.020	0.050
Family resilience → psychological distresss → social alienation	Indirect effect	−0.004 (−0.014, 0.001)	0.004	0.143
	Total indirect effect	−0.057 (−0.120, −0.011)	0.028	0.016
Peer support → social alienation	Total effect	−0.513 (−0.668, −0.354)	0.080	< 0.001
	Direct effect	−0.284 (−0.503, −0.044)	0.117	0.022
Peer support → psychological distress → social alienation	Indirect effect	−0.027 (−0.085, −0.001)	0.020	0.037
Peer support → negative social expectations → social alienation	Indirect effect	−0.178 (−0.334, −0.064)	0.068	0.002
Peer support → negative social expectations → psychological distress → social alienation	Indirect effect	−0.024 (−0.068, −0.002)	0.016	0.026
	Total indirect effect	−0.229 (−0.393, −0.105)	0.073	0.001

CI, Confidence Interval.

### Fuzzy-set qualitative comparative analysis results

To complement the SEM results and explore the complex interplay of conditions associated with social alienation, we performed fsQCA.

#### Data calibration

Following Ragin's calibration procedure,^24^ three anchor points were selected from the overall dataset for each condition. The scores corresponding to these anchor points for each variable are presented in Supplementary Table S2. Based on these anchors, the calibration function in fsQCA 3.0 was used to transform each sample into a membership score ranging from 0 to 1 within a fuzzy set (Supplementary Table S3).

#### Necessity analysis results

The necessity analysis revealed that no single condition achieved a consistency level above the 0.90 threshold for being a necessary condition for social alienation (all consistencies ≤ 0.76).^22^ This confirms that social alienation does not stem from any single condition but rather emerges from specific configurations of multiple conditions. Refer to Table 4.

**Table 4:** Necessary condition analysis for single factors.

Condition	High social alienation (CON)	High social alienation set (COV)	Absence of high social alienation (CON)	Absence of high social alienation set (COV)
Family communication and problem solving	0.51	0.52	0.71	0.75
∼Family communication and problem solving	0.76	0.72	0.53	0.55
Utilization of social and economic resources	0.67	0.59	0.72	0.65
∼ Utilization of Social and Economic Resources	0.60	0.68	0.55	0.63
Maintaining a positive outlook	0.58	0.55	0.73	0.70
∼Maintaining a positive outlook	0.68	0.71	0.53	0.57
Ability to make meaning of adversity	0.56	0.55	0.71	0.70
∼ Ability to Make Meaning of Adversity	0.70	0.70	0.55	0.56
Output expression	0.57	0.54	0.77	0.74
∼Output expression	0.73	0.75	0.56	0.53
Self-expression	0.57	0.55	0.77	0.75
∼Self-expression	0.74	0.76	0.54	0.56
Illness perception	0.54	0.56	0.69	0.73
∼Illness perception	0.74	0.70	0.58	0.56
Psychological distress	0.72	0.71	0.53	0.53
∼ Psychological Distress	0.53	0.52	0.71	0.72
Negative social expectations	0.76	0.77	0.49	0.51
∼ Negative Social Expectations	0.51	0.50	0.78	0.77

CON, Consistency; COV, Coverage. All values are rounded to two decimal places.

#### Truth table construction configurational analysis results

The raw consistency threshold was set to 0.8, the acceptable case threshold was set to 3, and the PRI consistency level was set to 0.7 ^36^^,^^37^(Supplementary Table S4).

The sufficiency analysis identified six distinct configurations that are consistently associated with high levels of social alienation. The overall solution consistency was 0.911, indicating that these configurations are highly consistent with the outcome. The overall solution coverage was 0.54.

As shown in Table 5, the six configurations can be categorized into two overarching patterns based on their core conditions: the Resource Deficiency-Negative Social Expectations Pattern (Configurations A1, A2, A3) and the Expression Deficiency-Psychological Distress Pattern (Configurations B1, B2, B3).

(1) Resource Deficiency-Negative Social Expectations Pattern: The core conditions unifying this pattern are the absence of utilization of social and economic resources and the presence of negative social expectations. This pattern characterizes patients who, lacking socioeconomic resources and holding pessimistic beliefs about their social worth, tend to exhibit high social alienation.
(2) Expression Deficiency-Psychological Distress Pattern: The core conditions for this pattern are the presence of psychological distress and a lack of expressive peer support. A common peripheral condition across these configurations is the co-occurrence of a maintained positive outlook and negative social expectations, indicative of a state of cognitive dissonance. This pattern highlights a link between emotional suffering coupled with an inability to express oneself to peers and higher social alienation, even when patients try to maintain a positive outlook.

**Table 5:** Configurational paths leading to social alienation in bladder cancer patients.

Condition variables and dimensions	Resource deficiency–Negative social expectations pattern			Expression deficiency–Psychological distress pattern		
	A1	A2	A3	B1	B2	B3
Family resilience						
Family communication and problem solving		⚪	⚪	⚪	●	●
Utilization of social and economic resources	♦	♦	♦		●	●
Maintaining a positive outlook	⚪		⚪	●	●	●
Ability to make meaning of adversity			⚪	●		⚪
Peer support						
Output expression	♦	♦	⚪	♦	♦	♦
Self-expression	⚪	⚪	⚪	⚪	⚪	⚪
Illness perception	⚪	⚪	⚪	⚪	⚪	⚪
Psychological distress	★	★		★	★	★
Negative social expectations	★	★	★	●	●	●
Raw coverage	0.324	0.317	0.316	0.221	0.187	0.176
Unique coverage	0.027	0.009	0.036	0.012	0.016	0.021
Consistency	0.959	0.951	0.96	0.951	0.958	0.952
Solution consistency	0.911					
Solution coverage	0.54					

★ *indicates that a core condition exists.* ●*: An auxiliary condition exists.* ♦*: The core condition is absent.* ⚪*: The auxiliary condition does not exist. Blank: This condition may or may not exist.*

#### Integration of SEM and fsQCA findings

SEM established net associations: psychological distress had a direct positive association with social alienation (Estimate = 0.137, *P* = 0.049), and peer support had a direct negative effect (Estimate = −0.284, *P* = 0.022). fsQCA extends these findings in two ways. First, psychological distress was not a necessary condition for high social alienation (consistency < 0.9). In the Expression Deficiency-Psychological Distress Pattern (Configurations B1–B3), psychological distress was associated with high alienation only when combined with a lack of expressive support (core absence of output expressive). Second, in the Resource Deficiency–Negative Social Expectations Pattern, psychological distress was a core condition in configurations A1 and A2, whereas configuration A3 did not specify its presence or absence. The core conditions in A3 were low utilization of social and economic resources and high negative social expectations. Thus, fsQCA revealed equifinal pathways, different combinations of conditions associated with the same outcome, that SEM's linear, additive model could not detect.

## Discussion

### Main findings

Guided by the CAPS theory, this study employed a dual-analytical approach to systematically investigate the complex factors and patterns associated with social alienation in BCa patients. By integrating symmetric (SEM) and asymmetric (fsQCA) techniques, the findings not only demonstrate the net associations of key predictors but, more importantly, support the intricate and equifinal patterns through which these factors combine to be associated with the outcome.

The SEM results provide the direct and indirect associations outlined in our model. Consistent with prior research in other populations,^7^^,^^17^ both family resilience and peer support were identified as significant interpersonal resources that are associated with lower levels of social alienation. Conversely, psychological distress and negative social expectations emerged as potent intrapersonal factors that are associated with higher levels of alienation. Beyond these direct associations, our findings illuminate the underlying psychological pathways. The significant indirect association of psychological distress in the paths from both family resilience and peer support to social alienation suggests that the quality of one's interpersonal context is linked to emotional well-being, which in turn is linked to one's engagement with the social world.^10^^,^^37^ Furthermore, the strong indirect association of negative social expectations between peer support and social alienation points to a critical cognitive factor. Inadequate support from peers may be connected with distorted beliefs about one's social worth and the expectations of others, creating a cognitive barrier that accompanies difficulties in social reconnection.^30^^,^^38^

The fsQCA results, however, reveal a more nuanced and complex picture that extends beyond the net associations captured by SEM. The identification of six sufficient configurations, coalescing into two distinct patterns, provides compelling evidence for equifinality, multiple, distinct pathways can be associated with the same outcome of high social alienation. The Resource Deficiency-Negative Social Expectations Pattern, characterized by the core absence of utilization of social and economic resources, and the presence of negative social expectations, highlights a particularly challenging state where practical inadequacies and pessimistic cognitions converge and are linked to social withdrawal.^39^^,^^40^ This pattern suggests that interventions solely focused on psychological support may be insufficient for this subgroup; instead, practical resource facilitation and cognitive restructuring are paramount.

The Expression Deficiency-Psychological Distress Pattern presents a more paradoxical profile. Here, the core presence of psychological distress and lack of expressive peer support co-occurs with the peripheral condition of maintaining a positive outlook alongside negative social expectations. This configuration may reflect optimism bias or “wishful thinking",^41^ where patients might consciously maintain a positive attitude while subconsciously harboring negative social fears, however, this interpretation remains speculative. This internal conflict, between their affirmed positivity and their unexpressed distress and social apprehensions, characterizes a state of cognitive dissonance that appears psychologically taxing and is associated with alienation.^42^ Importantly, this pattern may reflect a form of maladaptive coping involving emotional suppression rather than genuine psychological adjustment. Wu et al.^43^ demonstrated that cancer patients often engage in “emotional disguise”, concealing true negative emotions behind a pleasant external facade, a strategy associated with higher anxiety and family dysfunction. This suppression is not merely an individual choice but may be reinforced by the concept of “toxic positivity”,^44^ defined as the societal or self-imposed pressure to maintain a positive outlook at all costs, which invalidates authentic negative emotions and is associated with adverse psychological outcomes. Furthermore, Liao et al.^45^ identified “protective buffering” as a form of dyadic emotional labor in hematologic cancer patients, where individuals consciously conceal their struggles to shield family members from distress, thereby preserving relational harmony at the expense of authentic self-expression. Thus, the Expression Deficiency-Psychological Distress Pattern represents a convergence of these processes, where blocked authentic emotional expression is associated with higher social alienation. For these patients, interventions should therefore create safe spaces for genuine expression, through psychoeducation about toxic positivity and family-based communication training, rather than enforcing positivity.

Our integrated findings demonstrate the complementary value of combining SEM and fsQCA. While SEM confirmed the net positive association of psychological distress with social alienation, fsQCA revealed that this association is not universal but conditional. In configurations B1–B3 (the Expression Deficiency–Psychological Distress Pattern), psychological distress and low expressive peer support were both core conditions associated with high social alienation. In configuration A3 of the Resource Deficiency–Negative Social Expectations Pattern, the core conditions were low utilization of social and economic resources and high negative social expectations; psychological distress was unspecified. This equifinal finding challenges the implicit assumption of linear models that a single predictor (e.g., distress) is associated with the outcome uniformly across all patients. Instead, it suggests that for a subgroup of bladder cancer patients, alienation is linked to practical and cognitive deficits. Clinically, this suggests that patients presenting with the Resource Deficiency–Negative Social Expectations Pattern, social alienation may be more closely associated with resource deficits and negative social expectations than with psychological distress. Thus, the integration of variable-centered and case-centered methods offers preliminary insights that may inform personalized supportive care; however, these findings are hypothesis-generating and warrant prospective validation.

This study offers several significant contributions. Theoretically, this study applies the CAPS framework to social alienation in BCa patients, thereby supporting its utility as a framework for understanding the patterns of association between interpersonal contexts, cognitive-affective factors, and social alienation. Methodologically, this study illustrates the integration of SEM and fsQCA in the context of social alienation among BCa patients, which may also be applicable to similar behavioral research in nursing and psychology. The fsQCA findings, in particular, challenge simplistic linear assumptions and offer a more realistic, configurational understanding of patient experiences. From a practical perspective, our results provide actionable insights for developing targeted interventions. The SEM findings underscore the need for holistic care that addresses both interpersonal resources (e.g., strengthening family resilience through communication training, facilitating peer support groups) and intrapersonal factors (e.g., providing psychological counseling for distress, conducting cognitive-behavioral therapy to challenge negative expectations).^46^^,^^47^

The fsQCA results further enable precision-focused support. Future research could explore whether patients exhibiting the Resource Deficiency-Negative Social Expectations Pattern might respond to interventions that address practical needs, such as assistance navigating socioeconomic resources, or that target negative self-perceptions.^48^ For those exhibiting the Expression Deficiency-Psychological Distress Pattern, creating safe environments for emotional expression and using motivational interviewing and psychoeducation to help patients reconcile their conflicting thoughts and feelings, could help alleviate the distress associated with alienation . Ultimately, assessing a patient's specific profile across these conditions can guide health care providers in tailoring the most effective support strategy.

### Limitations

First, the cross-sectional design precludes causal inferences; longitudinal studies are needed. Second, convenience sampling from one Chinese hospital, self-report measures, and the predominantly older sample may limit generalizability and introduce bias, particularly for younger or employed patients. Third, unmeasured confounders (e.g., disease stage, treatment, personality) may still influence findings. Fourth, the SEM did not examine more complex serial mediation pathways. Fifth, the fsQCA results should be interpreted cautiously due to data-driven calibration, researcher-selected thresholds and directional expectations, limited diversity with nine conditions, and potential instability of configuration labels; future studies should cross-validate these configurations. Despite these limitations, this study delineates how multiple conditions combine to shape social alienation in patients with bladder cancer.

## Conclusions

This study identifies two distinct patterns linked to social alienation in bladder cancer patients: a Resource Deficiency-Negative Social Expectations Pattern, associated with a lack of socioeconomic resources and the presence of negative social expectations, and an Expression Deficiency-Psychological Distress Pattern, characterized by psychological distress and a lack of expressive peer support, often coexisting with a maintained positive outlook. These findings suggest that social alienation is characterized by specific combinations of conditions, not isolated factors. In clinical practice, this means health care professionals might identify patients with these profiles and implement tailored supportive care strategies.

## CRediT authorship contribution statement

**Yang Yixuan**: Methodology, Formal analysis, Writing – Original draft preparation. **Han Lu:** Software, Visualization. **Li Jie**: Writing – Review & Editing. **Xue Jun:** Supervision. **Wang Ting:** Investigation, Funding acquisition. **Zhao Qiuzhi:** Investigation. **Xie Cui:** Data curation. **Wang Jiaping:** Data curation. **Wang Xue:** Writing- Reviewing and Editing, Project administration. **Fu Li:** Conceptualization, Resources, Funding acquisition. All authors have read and approved the final manuscript.

## Ethics statement

This study was conducted in accordance with the Declaration of Helsinki and received ethical approval from the Medical Ethics Committee of the Second Hospital of Tianjin Medical University prior to data collection (Approval No. KY2023K223). All participants provided written informed consent.

## Data availability

The data that support the findings of this study are available from the corresponding author upon reasonable request. The data are not publicly available because of privacy or ethical restrictions.

## Declaration of generative AI and AI-assisted technologies in the writing process

No AI tools/services were used during the preparation of this work.

## Funding

This study was supported by the Science & Technology Development Fund of Tianjin Education Commission for Higher Education (Grant No. 2025KJ045). The funders had no role in considering the study design or in the collection, analysis, interpretation of data, writing of the report, or decision to submit the article for publication.

## Declaration of competing interest

The authors declare no conflict of interest.
