# Supplementary material for: Social alienation among people with bladder cancer: A combined structural equation modeling and fuzzy-set qualitative comparative analysis
Source: Asia Pac J Oncol Nurs. 2026 Sep 11;13:101025. doi: 10.1016/j.apjon.2026.101025 (PMC13632177; doi:10.1016/j.apjon.2026.101025)
Supplement: Supplementary file 1 [file mmc1.docx]

**Supplementary Table S1. Sensitivity analysis comparing structural equation models with and without covariate adjustment (N = 260, adjusted model controls for age and gender)**

| **Path** | **Unadjusted model** | | | **Adjusted model** | | |
| --- | --- | --- | --- | --- | --- | --- |
|  | **Std.Estimate** | **95%CI** | ***P*** | **Std.Estimate** | **95%CI** | ***P*** |
| Family Resilience→Psychological Distress | -0.081 | -0.287, -0.007 | 0.039 | -0.159 | -0.291, -0.014 | 0.031 |
| Family Resilience→Negative Social Expectations | -0.034 | -0.240, 0.023 | 0.096 | -0.112 | -0.241, 0.021 | 0.095 |
| Family Resilience→ Social Alienation | -0.152 | -0.239, -0.084 | 0.001 | -0.337 | -0.503, -0.167 | 0.001 |
| Negative Social Expectations→Psychological Distress | 0.440 | 0.082, 0.454 | 0.008 | 0.268 | 0.083, 0.454 | 0.008 |
| Negative Social Expectations→ Social Alienation | 0.441 | 0.105, 0.479 | 0.002 | 0.278 | 0.088, 0.418 | 0.004 |
| Psychological Distress→ Social Alienation | 0.148 | 0.033, 0.291 | 0.016 | 0.137 | 0.001, 0.276 | 0.049 |
| Peer Support→Psychological Distress | -0.254 | -0.395, -0.020 | 0.032 | -0.197 | -0.393, -0.018 | 0.033 |
| Peer Support→Negative Social Expectations | -0.499 | -0.642, -0.386 | 0.001 | -0.641 | -0.762, -0.503 | 0.001 |
| Peer Support→ Social Alienation | -0.307 | -0.487, -0.030 | 0.030 | -0.284 | -0.503, -0.044 | 0.022 |

**Supplementary Table S2. Calibration of the Outcome and Condition Variables**

| Type | Factors and Dimensions | Full Membership (95%) | Crossover Point (50%) | Full Non-Membership (5%) |
| --- | --- | --- | --- | --- |
| Outcome Variable | Social Alienation | 41 | 31 | 19 |
| Condition Variable | Family Communication and Problem-Solving | 101.95 | 92 | 80 |
|  | Utilization of Social and Economic Resources | 25 | 20 | 15 |
|  | Maintaining a Positive Outlook | 23 | 18 | 15 |
|  | Ability to Make Meaning of Adversity | 12 | 9 | 8 |
|  | Output Expression | 20 | 17 | 13 |
|  | Self-Expression | 25 | 20 | 11 |
|  | Illness Perception | 10 | 8 | 2 |
|  | Psychological Distress | 6 | 2 | 0 |
|  | Negative Social Expectations | 28 | 13 | 5 |

**Supplementary Table S3. Calibrated Membership Scores (Partial View of the Condition Variables)**

| ID | FCAPS | Calibrated Score | OE（DA） | Calibrated Score | PD(F) | Calibrated Score | NSE(e) | Calibrated Score |
| --- | --- | --- | --- | --- | --- | --- | --- | --- |
| 1 | 89 | 0.36 | 18 | 0.73 | 1 | 0.18 | 10 | 0.23 |
| 2 | 99 | 0.89 | 16 | 0.32 | 0 | 0.05 | 11 | 0.29 |
| 3 | 82 | 0.13 | 14 | 0.10 | 2 | 0.50 | 5 | 0.05 |
| 4 | 92 | 0.50 | 17 | 0.50 | 2 | 0.50 | 7 | 0.09 |
| 5 | 82 | 0.13 | 10 | 0.01 | 6 | 0.92 | 5 | 0.05 |
| 6 | 94 | 0.65 | 17 | 0.50 | 1 | 0.18 | 7 | 0.09 |
| 7 | 84 | 0.18 | 13 | 0.05 | 4 | 0.77 | 16 | 0.63 |
| 8 | 90 | 0.41 | 13 | 0.05 | 6 | 0.92 | 28 | 0.96 |
| 9 | 99 | 0.89 | 16 | 0.32 | 0 | 0.05 | 6 | 0.07 |
| 10 | 89 | 0.36 | 13 | 0.05 | 4 | 0.77 | 10 | 0.23 |
| 11 | 92 | 0.50 | 13 | 0.05 | 6 | 0.92 | 25 | 0.93 |
| 12 | 93 | 0.50 | 16 | 0.32 | 2 | 0.50 | 30 | 0.97 |
| 13 | 82 | 0.13 | 16 | 0.32 | 0 | 0.05 | 21 | 0.84 |
| 14 | 92 | 0.50 | 16 | 0.32 | 1 | 0.18 | 19 | 0.77 |
| 15 | 94 | 0.65 | 20 | 0.95 | 2 | 0.50 | 13 | 0.46 |
| 16 | 79 | 0.08 | 13 | 0.05 | 5 | 0.86 | 13 | 0.46 |
| 17 | 84 | 0.18 | 15 | 0.18 | 1 | 0.18 | 10 | 0.23 |
| 18 | 92 | 0.50 | 18 | 0.73 | 2 | 0.50 | 11 | 0.29 |
| 19 | 93 | 0.50 | 20 | 0.95 | 5 | 0.86 | 20 | 0.81 |
| 20 | 92 | 0.50 | 14 | 0.10 | 1 | 0.18 | 7 | 0.09 |
| 21 | 81 | 0.11 | 16 | 0.32 | 0 | 0.05 | 5 | 0.05 |
| 22 | 100 | 0.92 | 20 | 0.95 | 1 | 0.18 | 11 | 0.29 |
| 23 | 85 | 0.21 | 17 | 0.50 | 1 | 0.18 | 10 | 0.23 |
| 24 | 88 | 0.32 | 17 | 0.50 | 0 | 0.05 | 26 | 0.94 |
| 25 | 88 | 0.32 | 16 | 0.32 | 1 | 0.18 | 19 | 0.77 |
| 26 | 87 | 0.28 | 19 | 0.88 | 0 | 0.05 | 6 | 0.07 |
| 27 | 80 | 0.10 | 16 | 0.32 | 2 | 0.50 | 19 | 0.77 |
| 28 | 93 | 0.50 | 16 | 0.32 | 0 | 0.05 | 13 | 0.46 |
| 29 | 89 | 0.36 | 20 | 0.95 | 3 | 0.65 | 22 | 0.87 |
| 30 | 90 | 0.41 | 12 | 0.02 | 4 | 0.77 | 11 | 0.29 |
| 31 | 99 | 0.89 | 16 | 0.32 | 3 | 0.65 | 11 | 0.29 |
| 32 | 95 | 0.71 | 16 | 0.32 | 5 | 0.86 | 6 | 0.07 |
| 33 | 98 | 0.86 | 16 | 0.32 | 5 | 0.86 | 16 | 0.63 |
| 34 | 81 | 0.11 | 18 | 0.73 | 3 | 0.65 | 16 | 0.63 |
| 35 | 87 | 0.28 | 16 | 0.32 | 1 | 0.18 | 17 | 0.68 |
| 36 | 83 | 0.16 | 20 | 0.95 | 3 | 0.65 | 9 | 0.17 |
| 37 | 83 | 0.16 | 16 | 0.32 | 0 | 0.05 | 10 | 0.23 |
| 38 | 80 | 0.10 | 15 | 0.18 | 3 | 0.65 | 10 | 0.23 |
| 39 | 94 | 0.65 | 16 | 0.32 | 5 | 0.86 | 6 | 0.07 |
| 40 | 99 | 0.89 | 16 | 0.32 | 2 | 0.50 | 19 | 0.77 |
| 41 | 80 | 0.10 | 15 | 0.18 | 5 | 0.86 | 13 | 0.46 |
| 42 | 88 | 0.32 | 17 | 0.50 | 1 | 0.18 | 13 | 0.46 |
| 43 | 98 | 0.86 | 17 | 0.50 | 6 | 0.92 | 10 | 0.23 |
| 44 | 86 | 0.25 | 13 | 0.05 | 6 | 0.92 | 23 | 0.89 |
| 45 | 81 | 0.11 | 13 | 0.05 | 5 | 0.86 | 26 | 0.94 |
| 46 | 93 | 0.50 | 16 | 0.32 | 0 | 0.05 | 30 | 0.97 |
| 47 | 96 | 0.77 | 16 | 0.32 | 0 | 0.05 | 10 | 0.23 |
| 48 | 81 | 0.11 | 16 | 0.32 | 1 | 0.18 | 9 | 0.17 |
| 49 | 82 | 0.13 | 16 | 0.32 | 4 | 0.77 | 20 | 0.81 |
| 50 | 99 | 0.89 | 16 | 0.32 | 1 | 0.18 | 10 | 0.23 |
| 51 | 88 | 0.32 | 14 | 0.10 | 6 | 0.92 | 10 | 0.23 |
| 52 | 94 | 0.65 | 20 | 0.95 | 0 | 0.05 | 11 | 0.29 |
| 53 | 80 | 0.10 | 16 | 0.32 | 0 | 0.05 | 10 | 0.23 |
| 54 | 78 | 0.07 | 18 | 0.73 | 3 | 0.65 | 13 | 0.46 |
| 55 | 80 | 0.10 | 13 | 0.05 | 6 | 0.92 | 19 | 0.77 |
| 56 | 80 | 0.10 | 16 | 0.32 | 1 | 0.18 | 10 | 0.23 |
| 57 | 91 | 0.45 | 15 | 0.18 | 0 | 0.05 | 19 | 0.77 |
| 58 | 96 | 0.77 | 13 | 0.05 | 8 | 0.97 | 18 | 0.73 |
| 59 | 88 | 0.32 | 16 | 0.32 | 0 | 0.05 | 10 | 0.23 |
| 60 | 82 | 0.13 | 19 | 0.88 | 0 | 0.05 | 17 | 0.68 |
| 61 | 80 | 0.10 | 16 | 0.32 | 2 | 0.50 | 13 | 0.46 |
| 62 | 88 | 0.32 | 16 | 0.32 | 0 | 0.05 | 12 | 0.37 |
| 63 | 83 | 0.16 | 18 | 0.73 | 1 | 0.18 | 18 | 0.73 |
| 64 | 80 | 0.10 | 15 | 0.18 | 0 | 0.05 | 16 | 0.63 |
| 65 | 88 | 0.32 | 14 | 0.10 | 0 | 0.05 | 14 | 0.50 |
| 66 | 85 | 0.21 | 11 | 0.01 | 6 | 0.92 | 20 | 0.81 |
| 67 | 100 | 0.92 | 15 | 0.18 | 1 | 0.18 | 18 | 0.73 |
| 68 | 94 | 0.65 | 14 | 0.10 | 1 | 0.18 | 13 | 0.46 |
| 69 | 84 | 0.18 | 19 | 0.88 | 2 | 0.50 | 17 | 0.68 |
| 70 | 93 | 0.50 | 19 | 0.88 | 0 | 0.05 | 8 | 0.13 |
| 71 | 87 | 0.28 | 15 | 0.18 | 4 | 0.77 | 19 | 0.77 |
| 72 | 81 | 0.11 | 17 | 0.50 | 4 | 0.77 | 24 | 0.91 |
| 73 | 85 | 0.21 | 16 | 0.32 | 3 | 0.65 | 9 | 0.17 |
| 74 | 80 | 0.10 | 16 | 0.32 | 1 | 0.18 | 19 | 0.77 |
| 75 | 81 | 0.11 | 17 | 0.50 | 3 | 0.65 | 24 | 0.91 |
| 76 | 100 | 0.92 | 17 | 0.50 | 0 | 0.05 | 8 | 0.13 |
| 77 | 86 | 0.25 | 16 | 0.32 | 2 | 0.50 | 21 | 0.84 |
| 78 | 82 | 0.13 | 13 | 0.05 | 1 | 0.18 | 25 | 0.93 |
| 79 | 89 | 0.36 | 19 | 0.88 | 2 | 0.50 | 6 | 0.07 |
| 80 | 92 | 0.50 | 14 | 0.10 | 4 | 0.77 | 25 | 0.93 |
| 81 | 88 | 0.32 | 17 | 0.50 | 6 | 0.92 | 13 | 0.46 |
| 82 | 100 | 0.92 | 19 | 0.88 | 6 | 0.92 | 7 | 0.09 |
| 83 | 97 | 0.82 | 15 | 0.18 | 2 | 0.50 | 10 | 0.23 |
| 84 | 99 | 0.89 | 19 | 0.88 | 1 | 0.18 | 15 | 0.50 |
| 85 | 93 | 0.50 | 18 | 0.73 | 2 | 0.50 | 9 | 0.17 |
| 86 | 86 | 0.25 | 15 | 0.18 | 3 | 0.65 | 21 | 0.84 |
| 87 | 82 | 0.13 | 15 | 0.18 | 2 | 0.50 | 25 | 0.93 |
| 88 | 98 | 0.86 | 19 | 0.88 | 1 | 0.18 | 6 | 0.07 |
| 89 | 83 | 0.16 | 15 | 0.18 | 0 | 0.05 | 25 | 0.93 |
| 90 | 100 | 0.92 | 19 | 0.88 | 2 | 0.50 | 10 | 0.23 |
| 91 | 90 | 0.41 | 14 | 0.10 | 4 | 0.77 | 23 | 0.89 |
| 92 | 82 | 0.13 | 17 | 0.50 | 6 | 0.92 | 25 | 0.93 |
| 93 | 91 | 0.45 | 17 | 0.50 | 3 | 0.65 | 16 | 0.63 |
| 94 | 97 | 0.82 | 17 | 0.50 | 0 | 0.05 | 14 | 0.50 |
| 95 | 99 | 0.89 | 19 | 0.88 | 0 | 0.05 | 7 | 0.09 |
| 96 | 81 | 0.11 | 19 | 0.88 | 1 | 0.18 | 5 | 0.05 |
| 97 | 82 | 0.13 | 18 | 0.73 | 0 | 0.05 | 9 | 0.17 |
| 98 | 91 | 0.45 | 17 | 0.50 | 1 | 0.18 | 14 | 0.50 |
| 99 | 94 | 0.65 | 19 | 0.88 | 2 | 0.50 | 5 | 0.05 |
| 100 | 94 | 0.65 | 18 | 0.95 | 2 | 0.50 | 5 | 0.05 |

Note: FCAPS=Family Communication and Problem Solving; OE=Output Expression; PD=Psychological Distress; NSE = Negative Social Expectations.

**Supplementary Table S4: Truth Table Conditions**

| FCAPS | USER | MPO | AMMA | OE | SE | IP | NSE | PD | *N* | Row. | PRI. |
| --- | --- | --- | --- | --- | --- | --- | --- | --- | --- | --- | --- |
| 0 | 0 | 1 | 0 | 0 | 0 | 0 | 1 | 1 | 4 | 0.98 | 0.93 |
| 0 | 1 | 0 | 0 | 0 | 1 | 1 | 1 | 1 | 1 | 0.98 | 0.85 |
| 0 | 0 | 0 | 0 | 1 | 0 | 0 | 1 | 1 | 2 | 0.98 | 0.89 |
| 0 | 0 | 0 | 1 | 0 | 0 | 0 | 1 | 1 | 3 | 0.98 | 0.92 |
| 0 | 1 | 1 | 0 | 0 | 0 | 0 | 1 | 1 | 2 | 0.98 | 0.90 |
| 0 | 1 | 0 | 1 | 0 | 0 | 0 | 1 | 1 | 1 | 0.98 | 0.88 |
| 0 | 1 | 0 | 0 | 0 | 0 | 0 | 1 | 1 | 2 | 0.98 | 0.90 |
| 0 | 1 | 0 | 1 | 0 | 0 | 1 | 1 | 1 | 1 | 0.98 | 0.86 |
| 0 | 0 | 1 | 0 | 0 | 0 | 1 | 1 | 1 | 2 | 0.98 | 0.88 |
| 0 | 0 | 0 | 0 | 0 | 0 | 1 | 1 | 1 | 2 | 0.98 | 0.89 |
| 1 | 1 | 0 | 0 | 0 | 0 | 1 | 1 | 1 | 1 | 0.97 | 0.81 |
| 0 | 1 | 0 | 0 | 0 | 1 | 0 | 1 | 1 | 1 | 0.97 | 0.80 |
| 0 | 1 | 0 | 1 | 0 | 1 | 1 | 1 | 1 | 1 | 0.97 | 0.79 |
| 1 | 0 | 0 | 0 | 0 | 1 | 0 | 1 | 1 | 2 | 0.97 | 0.86 |
| 1 | 0 | 0 | 0 | 0 | 0 | 0 | 1 | 1 | 3 | 0.97 | 0.90 |
| 0 | 0 | 0 | 0 | 0 | 0 | 0 | 1 | 1 | 8 | 0.97 | 0.92 |
| 1 | 0 | 0 | 1 | 0 | 0 | 1 | 1 | 1 | 1 | 0.97 | 0.81 |
| 0 | 1 | 1 | 1 | 0 | 0 | 0 | 1 | 1 | 3 | 0.97 | 0.83 |
| 0 | 0 | 0 | 1 | 1 | 0 | 0 | 1 | 1 | 2 | 0.97 | 0.83 |
| 1 | 1 | 0 | 0 | 0 | 0 | 0 | 1 | 1 | 1 | 0.97 | 0.80 |
| 0 | 1 | 0 | 0 | 0 | 0 | 0 | 1 | 0 | 1 | 0.97 | 0.79 |
| 0 | 1 | 0 | 0 | 1 | 0 | 0 | 1 | 0 | 1 | 0.96 | 0.70 |
| 1 | 0 | 0 | 1 | 0 | 1 | 0 | 1 | 1 | 1 | 0.96 | 0.77 |
| 1 | 1 | 1 | 1 | 0 | 0 | 0 | 1 | 1 | 2 | 0.96 | 0.81 |
| 1 | 0 | 0 | 1 | 0 | 0 | 0 | 1 | 1 | 5 | 0.96 | 0.86 |
| 0 | 0 | 1 | 0 | 1 | 1 | 0 | 1 | 1 | 2 | 0.96 | 0.77 |
| 0 | 0 | 0 | 0 | 0 | 0 | 0 | 1 | 0 | 3 | 0.96 | 0.81 |
| 0 | 1 | 0 | 0 | 1 | 0 | 0 | 0 | 1 | 1 | 0.96 | 0.66 |
| 1 | 1 | 1 | 1 | 0 | 1 | 0 | 1 | 1 | 1 | 0.96 | 0.71 |
| 0 | 0 | 1 | 1 | 0 | 0 | 0 | 1 | 0 | 1 | 0.96 | 0.69 |
| 0 | 0 | 1 | 1 | 1 | 0 | 0 | 1 | 1 | 2 | 0.96 | 0.78 |
| 0 | 0 | 0 | 1 | 0 | 0 | 1 | 1 | 0 | 1 | 0.96 | 0.68 |
| 1 | 1 | 1 | 0 | 0 | 0 | 0 | 1 | 1 | 3 | 0.96 | 0.79 |
| 0 | 0 | 0 | 1 | 0 | 0 | 0 | 0 | 1 | 2 | 0.96 | 0.68 |
| 1 | 0 | 1 | 0 | 0 | 0 | 1 | 1 | 1 | 1 | 0.96 | 0.77 |
| 0 | 1 | 0 | 1 | 1 | 1 | 0 | 1 | 1 | 1 | 0.96 | 0.73 |
| 0 | 0 | 1 | 0 | 1 | 1 | 1 | 1 | 1 | 1 | 0.96 | 0.78 |
| 0 | 1 | 1 | 0 | 1 | 1 | 1 | 1 | 1 | 2 | 0.96 | 0.77 |
| 0 | 0 | 0 | 0 | 0 | 0 | 0 | 0 | 1 | 1 | 0.96 | 0.72 |
| 0 | 1 | 1 | 1 | 0 | 0 | 1 | 1 | 1 | 2 | 0.95 | 0.74 |
| 0 | 0 | 1 | 1 | 0 | 0 | 0 | 1 | 1 | 3 | 0.95 | 0.78 |
| 1 | 0 | 0 | 1 | 0 | 0 | 0 | 1 | 0 | 1 | 0.95 | 0.69 |
| 1 | 1 | 1 | 1 | 0 | 0 | 1 | 1 | 1 | 3 | 0.95 | 0.71 |
| 0 | 0 | 0 | 0 | 0 | 0 | 0 | 0 | 0 | 3 | 0.95 | 0.67 |
| 0 | 1 | 1 | 1 | 0 | 0 | 0 | 0 | 1 | 1 | 0.95 | 0.62 |
| 1 | 0 | 1 | 1 | 0 | 0 | 0 | 1 | 1 | 2 | 0.95 | 0.80 |
| 1 | 0 | 0 | 1 | 1 | 0 | 0 | 1 | 1 | 1 | 0.95 | 0.74 |
| 0 | 0 | 1 | 1 | 1 | 0 | 0 | 1 | 0 | 1 | 0.95 | 0.60 |
| 0 | 0 | 1 | 1 | 0 | 1 | 1 | 1 | 0 | 1 | 0.95 | 0.56 |
| 1 | 1 | 1 | 0 | 1 | 0 | 1 | 1 | 1 | 1 | 0.95 | 0.71 |
| 0 | 1 | 1 | 1 | 1 | 0 | 0 | 1 | 1 | 3 | 0.95 | 0.70 |
| 1 | 1 | 0 | 1 | 1 | 1 | 0 | 1 | 1 | 1 | 0.95 | 0.68 |
| 0 | 1 | 0 | 0 | 0 | 1 | 0 | 0 | 1 | 1 | 0.95 | 0.55 |
| 0 | 0 | 0 | 1 | 1 | 0 | 0 | 1 | 0 | 2 | 0.95 | 0.63 |
| 1 | 0 | 1 | 1 | 0 | 0 | 1 | 1 | 0 | 2 | 0.95 | 0.57 |
| 0 | 1 | 1 | 1 | 1 | 1 | 0 | 1 | 1 | 2 | 0.95 | 0.64 |
| 0 | 0 | 1 | 0 | 0 | 0 | 1 | 0 | 1 | 1 | 0.95 | 0.66 |
| 0 | 1 | 0 | 0 | 0 | 0 | 0 | 0 | 1 | 1 | 0.95 | 0.61 |
| 0 | 1 | 0 | 0 | 0 | 1 | 1 | 0 | 1 | 1 | 0.95 | 0.56 |
| 0 | 1 | 1 | 1 | 0 | 1 | 0 | 1 | 0 | 1 | 0.94 | 0.57 |
| 0 | 1 | 0 | 1 | 1 | 1 | 1 | 1 | 0 | 1 | 0.94 | 0.56 |
| 0 | 1 | 1 | 0 | 0 | 1 | 0 | 0 | 1 | 1 | 0.94 | 0.47 |
| 0 | 1 | 0 | 1 | 0 | 1 | 1 | 1 | 0 | 1 | 0.94 | 0.57 |
| 0 | 0 | 1 | 1 | 1 | 1 | 0 | 1 | 1 | 2 | 0.94 | 0.69 |
| 1 | 0 | 1 | 0 | 0 | 0 | 1 | 0 | 1 | 1 | 0.94 | 0.60 |
| 1 | 0 | 0 | 0 | 1 | 1 | 1 | 1 | 1 | 1 | 0.94 | 0.72 |
| 1 | 0 | 0 | 1 | 1 | 0 | 0 | 1 | 0 | 1 | 0.94 | 0.61 |
| 0 | 1 | 1 | 1 | 0 | 0 | 1 | 1 | 0 | 1 | 0.94 | 0.59 |
| 0 | 1 | 1 | 1 | 0 | 0 | 0 | 1 | 0 | 1 | 0.94 | 0.64 |
| 0 | 1 | 0 | 1 | 0 | 0 | 0 | 1 | 0 | 3 | 0.94 | 0.59 |
| 1 | 1 | 0 | 0 | 1 | 1 | 1 | 1 | 1 | 1 | 0.94 | 0.66 |
| 0 | 1 | 1 | 1 | 1 | 0 | 0 | 0 | 0 | 1 | 0.94 | 0.51 |
| 0 | 1 | 0 | 0 | 0 | 0 | 1 | 0 | 1 | 1 | 0.94 | 0.56 |
| 0 | 1 | 1 | 1 | 1 | 0 | 0 | 1 | 0 | 1 | 0.94 | 0.52 |
| 1 | 1 | 0 | 1 | 1 | 1 | 1 | 1 | 1 | 1 | 0.94 | 0.63 |
| 1 | 0 | 0 | 0 | 0 | 1 | 1 | 0 | 1 | 1 | 0.93 | 0.52 |
| 0 | 0 | 0 | 0 | 1 | 1 | 1 | 0 | 1 | 1 | 0.93 | 0.65 |
| 1 | 1 | 1 | 0 | 1 | 0 | 0 | 0 | 1 | 1 | 0.93 | 0.50 |
| 1 | 0 | 1 | 1 | 0 | 0 | 0 | 0 | 1 | 1 | 0.93 | 0.56 |
| 0 | 1 | 0 | 0 | 1 | 1 | 1 | 0 | 1 | 1 | 0.93 | 0.59 |
| 1 | 0 | 1 | 1 | 1 | 0 | 0 | 1 | 1 | 1 | 0.93 | 0.62 |
| 0 | 0 | 1 | 1 | 1 | 1 | 1 | 1 | 1 | 1 | 0.93 | 0.63 |
| 0 | 0 | 1 | 0 | 1 | 1 | 1 | 0 | 1 | 2 | 0.93 | 0.63 |
| 1 | 0 | 1 | 1 | 0 | 0 | 0 | 1 | 0 | 1 | 0.93 | 0.51 |
| 1 | 0 | 1 | 1 | 0 | 1 | 1 | 1 | 1 | 2 | 0.93 | 0.59 |
| 1 | 0 | 0 | 1 | 0 | 1 | 1 | 0 | 1 | 1 | 0.93 | 0.45 |
| 1 | 1 | 1 | 1 | 1 | 1 | 0 | 1 | 1 | 1 | 0.93 | 0.56 |
| 0 | 0 | 1 | 1 | 0 | 1 | 1 | 0 | 0 | 1 | 0.93 | 0.37 |
| 1 | 1 | 0 | 0 | 1 | 0 | 0 | 1 | 0 | 1 | 0.93 | 0.49 |
| 1 | 1 | 0 | 0 | 1 | 1 | 0 | 0 | 1 | 1 | 0.93 | 0.48 |
| 1 | 1 | 1 | 0 | 0 | 0 | 0 | 0 | 1 | 1 | 0.92 | 0.42 |
| 0 | 1 | 1 | 1 | 1 | 1 | 0 | 0 | 1 | 1 | 0.92 | 0.46 |
| 1 | 0 | 1 | 1 | 0 | 0 | 1 | 0 | 1 | 1 | 0.92 | 0.47 |
| 1 | 1 | 1 | 1 | 1 | 0 | 0 | 0 | 1 | 1 | 0.92 | 0.45 |
| 1 | 0 | 0 | 1 | 0 | 1 | 1 | 0 | 0 | 1 | 0.92 | 0.34 |
| 1 | 1 | 1 | 1 | 1 | 0 | 0 | 1 | 1 | 1 | 0.92 | 0.56 |
| 0 | 1 | 1 | 1 | 0 | 0 | 0 | 0 | 0 | 1 | 0.92 | 0.48 |
| 1 | 1 | 1 | 0 | 1 | 1 | 0 | 0 | 1 | 1 | 0.92 | 0.41 |
| 1 | 1 | 0 | 0 | 1 | 1 | 1 | 1 | 0 | 1 | 0.92 | 0.52 |
| 0 | 0 | 1 | 0 | 1 | 1 | 1 | 0 | 0 | 1 | 0.92 | 0.53 |
| 1 | 1 | 0 | 1 | 1 | 1 | 0 | 0 | 1 | 1 | 0.92 | 0.42 |
| 1 | 0 | 1 | 1 | 1 | 1 | 0 | 1 | 1 | 1 | 0.91 | 0.52 |
| 0 | 1 | 0 | 1 | 1 | 1 | 1 | 0 | 1 | 1 | 0.91 | 0.47 |
| 1 | 1 | 1 | 1 | 0 | 1 | 1 | 1 | 0 | 2 | 0.91 | 0.46 |
| 0 | 1 | 1 | 1 | 1 | 1 | 0 | 0 | 0 | 1 | 0.91 | 0.33 |
| 0 | 0 | 1 | 1 | 1 | 1 | 0 | 0 | 1 | 1 | 0.91 | 0.45 |
| 1 | 0 | 0 | 1 | 1 | 0 | 1 | 0 | 1 | 1 | 0.91 | 0.55 |
| 0 | 1 | 1 | 1 | 0 | 0 | 1 | 0 | 0 | 1 | 0.91 | 0.36 |
| 1 | 1 | 1 | 1 | 1 | 1 | 0 | 1 | 0 | 1 | 0.91 | 0.33 |
| 1 | 1 | 1 | 1 | 0 | 0 | 0 | 0 | 0 | 1 | 0.91 | 0.34 |
| 1 | 0 | 1 | 1 | 1 | 0 | 0 | 0 | 1 | 2 | 0.90 | 0.47 |
| 0 | 0 | 0 | 0 | 1 | 1 | 1 | 0 | 0 | 3 | 0.90 | 0.57 |
| 1 | 0 | 1 | 1 | 0 | 0 | 1 | 0 | 0 | 2 | 0.90 | 0.34 |
| 1 | 1 | 1 | 1 | 1 | 0 | 0 | 0 | 0 | 1 | 0.90 | 0.30 |
| 1 | 0 | 1 | 1 | 1 | 1 | 1 | 1 | 1 | 1 | 0.90 | 0.53 |
| 1 | 1 | 1 | 1 | 1 | 1 | 1 | 1 | 1 | 2 | 0.90 | 0.54 |
| 1 | 1 | 1 | 1 | 0 | 1 | 1 | 0 | 1 | 1 | 0.90 | 0.32 |
| 1 | 1 | 1 | 1 | 0 | 0 | 1 | 0 | 0 | 2 | 0.90 | 0.29 |
| 1 | 1 | 1 | 1 | 1 | 0 | 1 | 0 | 0 | 2 | 0.90 | 0.28 |
| 0 | 1 | 1 | 0 | 1 | 1 | 1 | 0 | 0 | 1 | 0.89 | 0.37 |
| 1 | 1 | 1 | 0 | 0 | 1 | 0 | 0 | 0 | 1 | 0.89 | 0.23 |
| 1 | 0 | 0 | 0 | 1 | 1 | 1 | 0 | 1 | 1 | 0.88 | 0.42 |
| 1 | 0 | 1 | 1 | 0 | 1 | 1 | 0 | 0 | 1 | 0.88 | 0.23 |
| 1 | 1 | 0 | 1 | 0 | 1 | 1 | 0 | 0 | 1 | 0.88 | 0.24 |
| 1 | 0 | 1 | 1 | 1 | 1 | 1 | 1 | 0 | 1 | 0.88 | 0.32 |
| 0 | 1 | 0 | 0 | 1 | 1 | 1 | 0 | 0 | 4 | 0.88 | 0.43 |
| 0 | 1 | 1 | 1 | 0 | 1 | 1 | 0 | 0 | 4 | 0.88 | 0.25 |
| 1 | 1 | 1 | 1 | 0 | 1 | 0 | 0 | 0 | 1 | 0.87 | 0.21 |
| 1 | 1 | 1 | 1 | 1 | 1 | 1 | 1 | 0 | 2 | 0.87 | 0.30 |
| 1 | 0 | 0 | 1 | 1 | 1 | 1 | 0 | 1 | 1 | 0.87 | 0.37 |
| 1 | 1 | 1 | 1 | 1 | 1 | 0 | 0 | 1 | 1 | 0.87 | 0.29 |
| 1 | 0 | 0 | 1 | 1 | 0 | 1 | 0 | 0 | 1 | 0.87 | 0.30 |
| 1 | 1 | 0 | 1 | 1 | 1 | 1 | 0 | 1 | 1 | 0.87 | 0.34 |
| 1 | 1 | 1 | 0 | 1 | 1 | 1 | 0 | 1 | 1 | 0.86 | 0.34 |
| 0 | 1 | 1 | 1 | 1 | 1 | 1 | 0 | 1 | 6 | 0.85 | 0.32 |
| 1 | 1 | 0 | 1 | 1 | 1 | 0 | 0 | 0 | 1 | 0.85 | 0.16 |
| 1 | 0 | 1 | 0 | 1 | 1 | 1 | 0 | 1 | 3 | 0.84 | 0.33 |
| 0 | 1 | 1 | 1 | 1 | 1 | 1 | 0 | 0 | 4 | 0.84 | 0.22 |
| 1 | 1 | 1 | 1 | 0 | 1 | 1 | 0 | 0 | 1 | 0.84 | 0.17 |
| 1 | 1 | 1 | 0 | 1 | 1 | 1 | 0 | 0 | 1 | 0.83 | 0.19 |
| 1 | 1 | 1 | 1 | 1 | 1 | 0 | 0 | 0 | 1 | 0.83 | 0.15 |
| 1 | 0 | 1 | 1 | 1 | 1 | 0 | 0 | 0 | 3 | 0.83 | 0.15 |
| 1 | 0 | 1 | 0 | 1 | 1 | 1 | 0 | 0 | 1 | 0.82 | 0.19 |
| 1 | 0 | 0 | 1 | 1 | 1 | 1 | 0 | 0 | 2 | 0.81 | 0.18 |
| 1 | 0 | 1 | 1 | 1 | 1 | 1 | 0 | 1 | 7 | 0.79 | 0.22 |
| 1 | 1 | 0 | 1 | 1 | 1 | 1 | 0 | 0 | 2 | 0.77 | 0.14 |
| 1 | 1 | 1 | 1 | 1 | 1 | 1 | 0 | 1 | 9 | 0.77 | 0.19 |
| 1 | 0 | 1 | 1 | 1 | 1 | 1 | 0 | 0 | 5 | 0.74 | 0.11 |
| 1 | 1 | 1 | 1 | 1 | 1 | 1 | 0 | 0 | 11 | 0.70 | 0.09 |

*Note: FCAPS= Family Communication and Problem Solving; USER= Utilization of Social and Economic Resources; MPO = Maintaining a Positive Outlook; AMMA= Ability to Make Meaning of Adversity; OE=Output Expression; SE= Self-expression; IP= Illness Perception; NSE = Negative Social Expectations; PD= Psychological Distress; N = Number of cases satisfying the configuration; Row. = Raw consistency; PRI. = PRI consistency score.*
